# Clinical course and long-term outcomes in autoimmune glial fibrillary acidic protein (GFAP) astrocytopathy

**DOI:** 10.1007/s00415-025-13159-0

**Published:** 2025-05-26

**Authors:** Joaquín Arzalluz-Luque, Pauline Dumez, Géraldine Picard, Marie Benaiteau, Maxime Bonjour, Pierre Lardeux, Julian Theuriet, Florian Lamblin, Marie Rafiq, Jerome Honnorat, Romain Marignier

**Affiliations:** 1 French Reference Center for Paraneoplastic Neurological Syndromes and Autoimmune Encephalitis, Hospices Civils de Lyon, Lyon, France; 2 Centre de Référence des Maladies Inflammatoires Rares du Cerveau et de la Moelle (MIRCEM), Service sclérose en plaques, pathologie de la myeline et neuro-inflammation, Hôpital Neurologique Pierre-Wertheimer, Hospices Civils de Lyon, 59 boulevard Pinel, 69677 Bron Cedex, France; 3 Department of Neurology, Hospital Universitario Virgen Macarena, 41009 Seville, Spain; 4 MeLis Institute, SynatAc Team, Inserm U1314/UMR CNRS5284, Lyon, France; 5 Service de biostatistique, Hospices Civils de Lyon, Lyon, France; 6 Service de Neuro-ophtalmologie, Neuro-otologie et neuro-cognition, Hôpital Neurologique Pierre-Wertheimer, Hospices Civils de Lyon, Bron, France; 7 Service d’ENMG et de pathologies neuromusculaires, centre de référence des maladies neuromusculaires PACA-Réunion-Rhône-Alpes, Hôpital Neurologique Pierre Wertheimer, Hospices Civils de Lyon, Bron, France; 8 Service de Neurologie, CHU de La Réunion, Saint-Pierre, France; 9 Service de Neurologie, Neuroscience Centre, Toulouse University Hospital, Toulouse, France; 10 Service de Neurologie, sclérose en plaques, pathologies de la myéline et neuro-inflammation, Hôpital Neurologique Pierre-Wertheimer, Hospices Civils de Lyon, Bron, France

**Keywords:** GFAP astrocytopathy, Neuroimmunology, Autoimmune encephalitis, Myelitis, MRI

## Abstract

**Background:**

The aim was to describe the clinical course and long-term outcomes of the French cohort of patients with glial fibrillary acidic protein (GFAP) astrocytopathy.

**Methods:**

Patients with positive CSF GFAP antibody test were identified between May 2017 and February 2023. Those whose clinical presentation occurred < 2 years before the initiation of the study, with other diagnosis than GFAP astrocytopathy, and with missing clinical information were excluded.

**Results:**

Among the 74 patients included, 71 were alive at last follow-up. The median age at onset was 43 years (range 6–84), 44 patients were male (62%), and 11 (15%) had a neoplasia. The main initial syndrome was meningo-encephalitis (*n* = 41, 58%). The median follow-up was 28 months (range 1–129). The median mRS at presentation was 4 (range 1–5) and at last follow-up was 1 (range 0–4). Forty patients reported disability at last follow-up (56%). The most frequent sequelae were cognitive complaints (20/40, 50%) and gait disorder (19/40, 48%). 38/55 patients (69%) returned to school/work. Long-term immunoactive treatment was introduced in 40 patients (56%); the most commonly administered were oral corticosteroids (*n* = 35, 49%). Relapses were documented in 10 patients (14%) and occurred after a median follow-up of 9 months (range 3–46). The presence of concomitant tumor at onset was associated with relapse (HR 4.55, 95% CI 1.28–16.14, *p* = 0.03).

**Conclusions:**

This study suggests a greater impact than previously described in long-term outcomes of patients with GFAP astrocytopathy and reports concomitant tumor at presentation as a risk factor for relapse.

**Supplementary Information:**

The online version contains supplementary material available at 10.1007/s00415-025-13159-0.

## Introduction

Autoimmune glial fibrillary acidic protein (GFAP) astrocytopathy is a rare and recently reported entity, characterized by the presence of GFAP autoantibodies (GFAP-Abs) in the CSF [1]. It has been mainly described as a meningo-encephalitis in isolation or associated with other symptoms/signs, such as myelitis [1], or optic nerve [2], or peripheral nervous system (PNS) involvement [3]. This clinical picture is often accompanied by abnormal MRI (brain linear perivascular radial enhancement is a hallmark of the disease) [4, 5], and inflammatory CSF findings [6, 7]. The cause of autoimmune GFAP astrocytopathy is still unknown but infectious triggers, the co-existence of tumor, and association with human leucocyte antigen (HLA) loci have been reported [8]. The disease is often monophasic and highly responsive to corticosteroids. Nevertheless, a relapsing course, worsening with corticosteroid withdrawal, and need for second-line immunotherapies have been reported, but also very severe course and fatal cases are rare but possible [9, 10]. Several national cohort studies have been published since its first description [11, 14]. However, long-term evaluation and prognosis factors are still missing. The aim of this study was to describe the clinical course and long-term outcomes of the French cohort of patients with autoimmune GFAP astrocytopathy, in terms of clinical, radiological and laboratory features, and treatment strategy.

## Methods

### Study participants

In the present observational study, we reviewed the databases of the French Reference Center for Rare Brain and Spinal Cord Inflammatory Diseases (MIRCEM), and the French National Reference Center for Autoimmune Encephalitis in Lyon, France, and we identified patients with a positive CSF GFAP immunoglobulin G (IgG) antibody test, as previously described [8], between May 2017 and February 2023. First, we excluded patients whose clinical presentation occurred < 2 years before the initiation of the study (i.e., February 2021), in order to select patients with a long follow-up. We also excluded patients who had a diagnosis other than GFAP astrocytopathy, as well as those with missing clinical information. Those included had GFAP astrocytopathy and initial presentation before February 2021. Deceased patients were analyzed separately. Among patients alive at last follow-up, relapsing patients were also analyzed as a sub-group.

### Data collection

Data of interest were collected retrospectively from electronic reports and anonymized. At initial presentation, age at first episode and sex, as well as any history of tumor, the result of brain and spinal cord MRI, and initial CSF analyses (protein levels, white cell count, glucose levels, the presence of oligoclonal bands [OCB] ≥ 2, and positive CSF GFAP-IgG) were collected. For each relapse, clinical presentation, modified Rankin Scale (mRS), intensive care unit admission, orotracheal intubation, time to relapse, and initiation of long-term treatment were collected. Long-term treatments were defined as > 3 months of oral corticosteroids or monthly intravenous immunoglobulin (IVIg) infusions, or azathioprine, mycophenolate mofetil (MMF), rituximab, or cyclophosphamide. Longitudinal assessment included follow-up duration, disability (change in mRS over time, functional systems affected, and ability to return to work/school), long-term treatment and the results of follow-up MRI, CSF analysis, and CSF GFAP-IgG status, which were collected, as was chemotherapy for associated tumor. Follow-up MRIs were grouped into three categories compared to features at onset (improvement, stabilization, or worsening), as well as for follow-up CSF.

### Statistical analyses

Descriptive statistics were reported as frequencies and percentages for categorical variables and as median and range for quantitative variables. The associations between the risk of relapse and the clinical, imaging and laboratory features at onset were assessed with odds ratio (OR) for categorical data, and a two-tailed Fisher exact test to estimate its statistical significance. For quantitative variables, the mean differences were assessed according to the risk of relapse with Student *t* and Mann–Whitney *U* tests (according to test assumptions). The association of clinical, imaging and laboratory features at onset with the time to relapse was evaluated according to the Kaplan–Meier method; comparisons of survival distributions were made with the non-parametric log-rank test. Hazard ratios (HR) were calculated along with their 95% confidence interval (CI) using univariate Cox regression analyses. *P* values < 0.05 were considered significant. Statistical analyses were conducted using IBM SPSS statistics (version 28.0.1.0), as well as R software (version 2023.06.2 + 561; R Core Team 2023).

### Standard protocol approvals, registrations and patient consents

The study was approved by the institutional review board of the Hospices Civils de Lyon (19–308) and was performed in accordance with the ethical standards laid down in the 1964 Declaration of Helsinki and its later amendments. All patients received oral and written information, and written informed consent was obtained.

## Results

### Cohort description

**Fig. 1:**
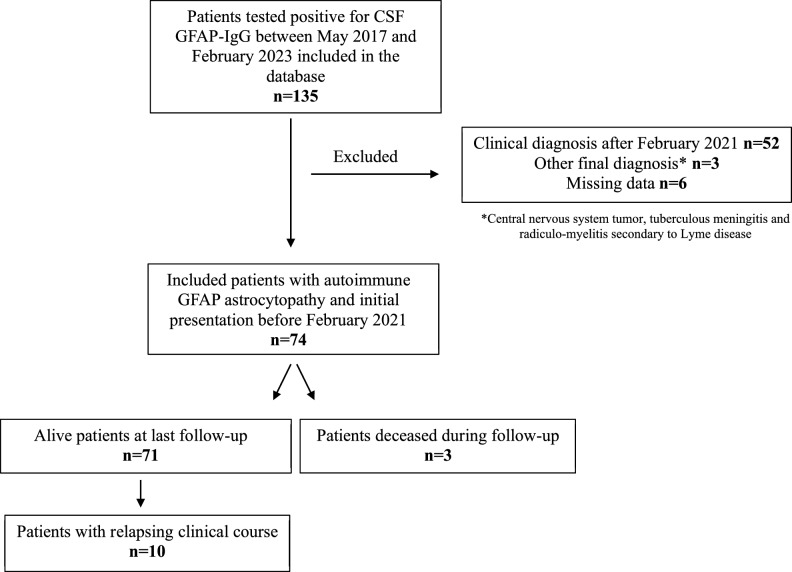
Flowchart of included patients in the study

Among the 135 patients tested positive for CSF GFAP-IgG in the database between May 2017 and February 2023, 52 had a clinical diagnosis after February 2021, 3 had a final diagnosis that was not GFAP astrocytopathy (central nervous system tumor, tuberculous meningitis, and radiculo-myelitis secondary to Lyme disease), and 6 were excluded for missing clinical data. 74 patients were included; 71 patients were alive at last follow-up and 3 patients died during follow-up (Fig. 1). Among the 3 deaths, 2 were related to GFAP astrocytopathy and 1 was not. The median age of deceased patients was 63 (range 41–72). Data concerning deceased patients are presented as Supplemental Material. Autoimmune GFAP astrocytopathy-related deaths occurred in the context of severe presentations. These patients were admitted to an intensive care unit and received orotracheal intubation, and deteriorated progressively, leading to death despite intensive care at, respectively, 2 and 15 months of follow-up. The third patient had metastatic renal cancer and died 15 months after diagnosis from status epilepticus and respiratory complications secondary to brain metastasis.

### Demographic, clinical, imaging, and laboratory features

The median age at onset was 43 years (range 6–84 years), and most patients were male (*n* = 44, 62%). Eleven patients (15%) had a history of neoplasia (4/11 lung adenocarcinoma, 3/11 ovarian teratoma, 3/11 renal cancer, and 1/11 lymphoma). At onset, clinical syndrome was meningo-encephalitis in 41 patients (58%), meningo-encephalo-myelitis in 17 (24%), meningitis in 10 (14%), myelitis in 2 (3%), and meningoradiculitis in 1 patient (1%). Sixteen patients (23%) had additional optic nerve involvement, and 21 (30%) had PNS involvement. Twenty-two patients (31%) were admitted to an intensive care unit, and 10 patients (14%) underwent orotracheal intubation. The median mRS at initial presentation was 4 (range 1–5); 1 patient (1%) had mRS < 2, 14 (20%) had mRS = 2, 13 (18%) had mRS = 3, 17 (24%) had mRS = 4, and 26 patients (37%) had mRS = 5 (Table 1).

**Table 1:** Clinical, imaging and laboratory characteristics at onset

	Alive patients at last follow-up *N* = 71
Male sex, *n* (%)	44 (62)
Age (years), median (range)	43 (6–84)
Pediatric patients (< 18 years), *n* (%)	10 (14)
Tumor, *n* (%)^a^	11 (15)
Clinical syndrome at onset:	
Meningoencephalitis, *n* (%)	41 (58)
Meningo-encephalo-myelitis, *n* (%)	17 (24)
Meningitis, *n* (%)	10 (14)
Myelitis, *n* (%)	2 (3)
Meningoradiculitis, *n* (%)	1 (1)
Additional clinical features:	
Optic nerve involvement, *n* (%)	16 (23)
PNS involvement, *n* (%)	21 (30)
ICU admission, *n* (%)	22 (31)
Orotracheal intubation, *n* (%)	10 (14)
Maximum mRS score, median (range)	4 (1–5)
< 2, *n* (%)	1 (1)
2, *n* (%)	14 (20)
3, *n* (%)	13 (18)
4, *n* (%)	17 (24)
5, *n* (%)	26 (37)
Abnormal initial brain MRI, *n* (%)	46 (65)
Abnormal initial spinal cord MRI, *n* (%)	24/42 (57)
Initial CSF analysis	
Elevated protein level (> 45 mg/dL), *n* (%)	66/69 (96)
Protein level (mg/dL), median (range)	120 (24–500)
Elevated white cell count (> 5/μL), *n* (%)	71 (100)
White cell count (cell/μL), median (range)	270 (6–3740)
Low glucose level (< 40 mg/dL), *n* (%)	5/63 (8)
OCB (≥ 2), *n* (%)	33/43 (77)

*CSF* cerebrospinal fluid, *ICU* intensive care unit, *MRI* magnetic resonance imaging, *mRS* modified Rankin scale, *OCB* oligoclonal bands, *PNS* peripheral nervous system

^a^Tumors found: 4/11 lung adenocarcinoma, 3/11 ovarian teratoma, 3/11 renal cancer and 1/11 lymphoma

Initial brain MRI was abnormal in 46 patients (65%), and initial spinal cord MRI was abnormal in 24/42 (57%; Table 1). Regarding brain MRI, 37 patients (52%) had abnormal T2-weighted/fluid-attenuated inversion recovery (FLAIR) hyperintensities, and 31 patients (44%) had gadolinium enhancements. T2 hyperintensities were multifocal in 23 (32%); these involved supratentorial areas in 27 (38%) patients and infratentorial areas in 13 (18%). Two patients had optic nerve lesions on MRI (3%). Leptomeningeal gadolinium enhancement was found in 23 patients (32%), and perivascular radial enhancement was found in 13 patients (18%). Other enhancement patterns included patchy (*n* = 5, 7%), cranial nerve involvement (*n* = 5, 7%) and periependymal (*n* = 2, 3%). Restricted diffusion was observed in 10/40 (25%), including 9 patients with splenial corpus callosum lesions. On spinal cord MRI, T2 hyperintensities were observed in 18/42 patients (43%), with 9/42 patients (21%) having longitudinally extensive spinal cord lesions (≥ 3 vertebral segments long). Lesions were located in the cervical spinal cord in 13 (31%) patients, thoracic spinal cord in 9 (21%), and lumbar spinal cord in 3 (7%), with multifocal distribution in 6 patients (14%). Gadolinium enhancement was found in 17/42 (40%) patients: leptomeningeal, 12 (29%); patchy, 4 (10%); and periependymal, 2 (5%).

Initial CSF analysis found a median protein level of 120 mg/dL (range 24–500 mg/dL) as well as an elevated white cell count in all patients and a median of 270 cells/μL (range 6–3740 cells/μL). A low glucose level (< 40 mg/dL) was found in 5/63 patients (8%), and OCB were found in 33/43 patients (77%; Table 1).

### Outcome and follow-up

**Fig. 2:**
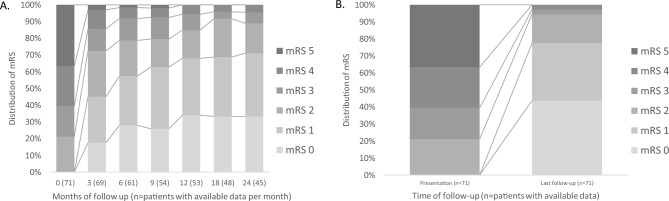
Evolution of disability (mRS) with long-term follow-up for the overall cohort. Within both figures, abscissa axis indicates time (months of follow-up in (**A**) and presentation vs. last follow-up in (**B**)) and the number of patients with available data, whereas ordinate axis represents the percentage of patients with a certain mRS score. Data in **A** are shown for the first 24 months of follow-up. Data in **B** include the overall cohort at presentation and last follow-up. *mRS* modified Rankin scale

The median follow-up was 28 months (range 1–129 months). At last follow-up, the median mRS was 1 (range 0–4); 31 patients (43%) had mRS = 0, 24 (34%) had mRS = 1, 12 (17%) had mRS = 2, 2 patients (3%) had mRS = 3, and 2 patients (3%) had mRS = 4 (Table 2). How disability (mRS) evolved over time with long-term follow-up for the overall cohort is presented in Fig. 2. Forty patients reported disability at last follow-up (40, 56%). The most frequent sequelae were cognitive complaints (20/40, 50%) and gait disorder (19/40, 48%). 38/55 patients (69%) were able to return to school/work (Table 2). Cognitive complaints affected predominantly executive functions (11/20, 55%), whereas episodic memory (7/20, 35%), language (1/20, 5%), and behavioral disorder (1/20, 5%) were less frequent. Gait disorders were mostly due to cerebellar dysfunction (7/19, 37%) or sensory ataxia (5/19, 26%); also related to motor deficit (7/19, 37%) and fatigue (2/19, 11%). Other sequelae reported were sphincter disorders (11/40, 28%), visual impairment (3/40, 8%), sensory symptoms (2/40, 5%), and seizures (2/40, 5%; Table 2).

**Table 2:** Clinical, imaging, laboratory and treatment characteristics at last follow-up

	Alive patients at last follow-up *N* = 71
Follow-up (months), median (range)	28 (1–129)
mRS, median (range)	1 (0–4)
0, *n* (%)	31 (43)
1, *n* (%)	24 (34)
2, *n* (%)	12 (17)
3, *n* (%)	2 (3)
4, *n* (%)	2 (3)
≥ 5, *n* (%)	–
Disability, *n* (%)	40 (56)
Cognitive complaints, *n* (%)	20/40 (50)
Gait disorder, *n* (%)	19/40 (48)
Sphincter disorder, *n* (%)	11/40 (28)
Visual impairment, *n* (%)	3/40 (8)
Sensory symptoms, *n* (%)	2/40 (5)
Seizures, *n* (%)	2/40 (5)
Return to work/school^a^, *n* (%)	38/55 (69)
Time to follow-up MRI (months), median (range)	11 (1–47)
Brain MRI	
Improvement, *n* (%)	40/52 (77)
Stabilization, *n* (%)	8/52 (15)
Worsening, *n* (%)	4/52 (8)
Spinal cord MRI	
Improvement, *n* (%)	19/22 (86)
Stabilization, *n* (%)	3/22 (14)
Worsening, *n* (%)	–
Time to follow-up CSF analysis (months), median (range)	7 (2–47)
CSF protein	
Normalization, *n* (%)	47/49 (96)
Worsening, *n* (%)	2/49 (4)
CSF white cell count	
Normalization, *n* (%)	48/49 (98)
Worsening, *n* (%)	1/49 (2)
CSF OCB (≥ 2), *n* (%)	23/32 (72)
Time to follow-up CSF GFAP-IgG analysis (months), median (range)	8 (1–49)
CSF GFAP-IgG positive analysis, *n* (%)	17/29 (59)
Long-term treatment, *n* (%)	40 (56)
Oral corticosteroids > 3 months, *n* (%)	35 (49)
Rituximab, *n* (%)	8 (11)
IVIg > 3 months, *n* (%)	5 (7)
Azathioprine, *n* (%)	2 (3)
Mycophenolate mofetil, *n* (%)	2 (3)
Cyclophosphamide, *n* (%)	2 (3)
Chemotherapy, *n* (%)	2 (3)

*CSF* cerebrospinal fluid, *GFAP* glial fibrillary acidic protein, *IgG* immunoglobulin G, *IVIg* intravenous immunoglobulin, *MRI* magnetic resonance imaging, *mRS* modified Rankin scale, *OCB* oligoclonal bands

^a^Equivalent to recover to complete autonomy if retired patients

Last follow-up brain MRI was available for 52 patients after a median follow-up of 11 months (range 1–47 months); this found improvement in 40/52 (77%), stabilization in 8/52 (15%), and worsening in 4/52 (8%). Among these 4 patients whose MRI deteriorated, 2 were relapsing. One patient had no disability (mRS 0) at last follow-up, whereas the 3 other patients had: mRS 1 (sphincter disorder), mRS 2 (cognitive complaints and seizures) and mRS 3 (gait disorder). Follow-up spinal cord MRI was available for 22 patients: 19/22 (86%) improved, and 3/22 (14%) were stable (Table 2).

CSF analysis was assessed again in 49 patients during follow-up, after a median of 7 months (range 2–47). CSF protein level normalized in 47/49 (96%) as did the CSF white cell count in 48/49 (98%). In 1 patient, the worsening of protein and cell count level in the CSF occurred in the setting of a relapse, while for the other patient, the rise in CSF protein level was due to an early follow-up lumbar puncture after 1 month from onset. CSF OCB were positive in 23/32 (72%), among whom 20/23 (87%) were persistent, and 3/23 (13%) were positive results in previously not tested patients; 9/32 (28%) patients had negative OCB, since OCB remained negative in 5/9 (56%) patients and turned negative in 4/9 (44%) patients. Twenty-nine patients were tested again for CSF GFAP-IgG, and 17/29 (59%) were still positive (Table 2).

### Treatment

Long-term maintenance immunoactive treatment was introduced in 40 patients (56%): 35 patients (49%) received oral corticosteroids for > 3 months, 8 (11%) had courses of rituximab, 5 (7%) monthly infusions of IVIg for > 3 months, 2 (3%) received azathioprine, 2 (3%) MMF, 2 (3%) infusions of cyclophosphamide, and 2 (3%) chemotherapy for associated neoplasia (Table 2).

### Characteristics and predictors of relapse

Relapsing course was documented in 10 patients (10/71, 14%) during a median follow-up of 28 months (range 1–129 months). These patients had a median age of 41 years (range 16–72 years). Relapses occurred after a median follow-up of 9 months (range 3–46 months; Table 3).

**Table 3:** Demographic and clinical characteristics of patients with relapsing course

Patient	Sex, M/F	Tumor	Clinical presentation				Relapses					Follow-up, months	mRS at last follow-up	Disability cause
			Syndrome	mRS	ICU	Long-term treatments	Syndrome	Time to relapse, months	mRS	ICU	Long-term treatments			
No. 1	M	Lung ADK	Meningoencephalitis	2	Yes (agitation) No intubation	OCS taper	Meningoencephalitis + bilateral optic neuropathy	6	2	No	Azathioprine	57	1	Non-disabling mild cerebellar ataxia
No. 2	M	No	Meningoencephalitis	3	No	IVIg (monthly, 3 months) OCS taper	Meningo-encephalo-myelitis	11	4	No	Rituximab OCS taper (19 months)	32	0	-
No. 3	M	No	Meningo-encephalo-myelitis	5	No	OCS taper (19 months) IVIg (monthly, 14 months)	Meningoencephalitis	23	4	No	OCS taper restarted IVIg restarted (time not known) Rituximab	33	3	Cerebellar syndrome affecting walk, daily activities and work
No. 4	F	No	Meningo-encephalo-myelitis and bilateral optic neuropathy	4	No	OCS taper Rituximab	1. Bilateral optic neuropathy	7	3	No	OCS increased Rituximab maintained	48	1	Phosphenes. Normal neurological and ophthalmological exam
							2. Right optic neuropathy	15	3	No	OCS increased (time not known) Rituximab maintained			
No. 5	F	Ovarian teratoma	Meningitis	2	No	No	Myelitis	3	3	No	MMF	70	1	Sensory symptoms
No. 6	F	No	Meningoencephalitis	4	No	OCS taper	Meningoencephalitis	20	4	No	OCS taper continued (5 months)	38	4	Severe cerebellar ataxia and sphincter disorder
No. 7	M	No	Meningoencephalitis	2	Yes, without intubation	No treatment, spontaneous improvement	Meningoencephalitis	16	1	No	OCS taper (time not known)	36	0	-
No. 8	F	Ovarian teratoma	Meningoencephalitis	5	Yes, without intubation	Teratoma surgery OCS taper Rituximab (1 dose)	Meningoencephalitis	4	4	No	OCS taper (4 months) Cyclophosphamide (monthly, 6 months)	26	4	Cognitive and gait impairment
No. 9	F	No	Meningo-encephalo-myelitis	3	Yes, without intubation	OCS taper (3 months)	Meningoencephalitis	46	1	No	OCS taper restarted (3 months) Rituximab (1 dose)	55	0	-
No. 10	M	Renal cancer + Nivolumab	Meningoencephalitis	3	No	OCS taper Nivolumab stopped	Encephalitis	3	3	No	OCS higher dose and taper (3 months)	12	1	Epilepsy and mild cognitive impairment

*ADK* adenocarcinoma, *F* female, *ICU* intensive care unit, *IVIg* intravenous immunoglobulin, *M* male, *MMF* mycophenolate mofetil, *mRS* modified Rankin scale, *OCS* oral corticosteroids

At initial presentation, 80% (8/10) of relapsing patients received long-term treatment. Among those treated, all received oral corticosteroids, with additional IVIg in 2 cases and concomitant rituximab in 2 other cases. After relapse, all patients were treated. Three patients (3/10, 30%) were treated with corticosteroids and 7 patients (7/10, 70%) received second-line immunoactive treatment: rituximab (4/7), cyclophosphamide (1/7), azathioprine (1/7), or MMF (1/7). Among the 2 who initiated rituximab from the onset, 1 maintained rituximab and the other switched to cyclophosphamide after the relapse. Regarding the 2 patients who were not treated at initial presentation, 1 received oral corticosteroids and the other MMF (Table 3).

Overall, most of the relapsing patients (7/10, 70%) had a final mRS between 0 and 1. However, three patients (3/10, 30%) had mRS > 1 despite long-term treatment: mRS 3 (gait disorder), mRS 4 (gait and sphincter disorder), and mRS 4 (gait and cognitive disorder; Table 3).

Four of the relapsing patients had a history of tumor: lung adenocarcinoma, renal cancer treated with nivolumab, and 2 ovarian teratoma. Disease control was achieved for patient No.1 after the discovery and treatment of a lung adenocarcinoma and initiation of azathioprine at the time of relapse at the 6 th month. Patient No.5 was not initially treated but received MMF after the 2nd episode at the 3rd month of follow-up in addition to cancer treatment. The initial clinical presentation of patient No.8 was severe, had a mRS = 5, and was admitted to an intensive care unit. Rituximab and corticosteroids were introduced from the start and she underwent urgent surgery to remove the teratoma. However, she relapsed at the 4 th month and, although she was treated with cyclophosphamide, she remained disabled (mRS = 4) after 26 months of follow-up (cognitive and gait impairment). As for patient No. 10, autoimmune GFAP astrocytopathy appeared in the setting of renal cancer and treatment with nivolumab, which was discontinued. Oral corticosteroids’ tapering was introduced, and doses were increased after the 2nd episode 3 months later. Other than patient No.8, relapsing patients with cancer showed no relevant disability at last follow-up (mRS = 1; Table 3).

**Fig. 3:**
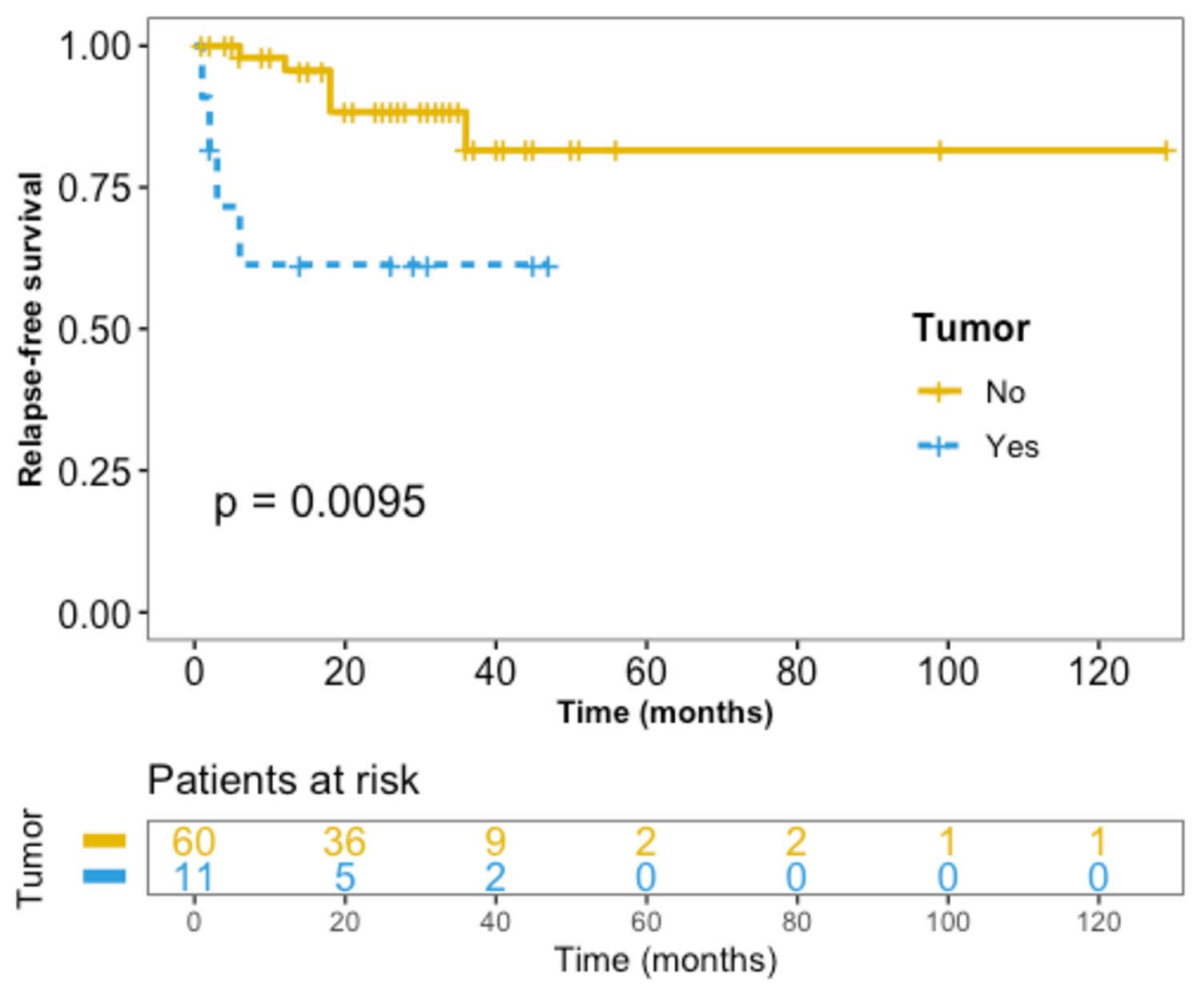
Kaplan–Meier survival analysis for relapse stratified according to the presence of tumor at onset. Yellow line represents patients without a tumor and blue line represents patients with a tumor at onset. Log-rank test *p* = 0.0095. HR 4.55, 95% CI 1.28–16.14, *p* = 0.03

Univariate analysis of the association of onset characteristics with relapse found that the presence of a tumor at the onset of neurological symptoms was associated with relapse (OR 5.14, 95% CI 1.16–22.82, p = 0.042; Table 4). Regarding survival analysis, similarly, the presence of a tumor at onset was associated with relapse (HR 4.55, 95% CI 1.28–16.14, p = 0.03; Table 5). Kaplan–Meier survival analysis for relapse stratified according to the presence of tumor at onset is presented in Fig. 3. No other baseline characteristic showed statistical significance as predictor of relapse, neither did the persistency of CSF GFAP-IgG (Tables 4 and 5).

**Table 4:** Univariate analysis of the association of onset characteristics with relapse

	Alive patients at last follow-up *N* = 71		*p* value	OR [95% CI]
	Relapsing *N* = 10	Non-relapsing *N* = 61		
Male sex, *n* (%)	5 (50)	39 (64)	0.49	1.76 [0.36–8.58]
Age (years), median (range)	41 (16–72)	46 (6–84)	0.77	–
Age ≤ 43 years^a^, *n* (%)	5 (50)	33 (54)	1	1.18 [0.24–5.69]
Tumor, *n* (%)	4 (40)	7 (12)	**0.042**	**5.14 [1.16–22.82]**
Clinical syndrome at first episode:				
Meningoencephalitis, *n* (%)	6 (60)	35 (57)	1	1.11 [0.24–5.93]
Meningo-encephalo-myelitis, *n* (%)	2 (20)	15 (25)	1	0.77 [0.07–4.49]
Meningitis, *n* (%)	2 (20)	8 (13)	0.62	1.64 [0.15–10.66]
Myelitis, *n* (%)*	–	2 (3)	1	–
Meningoradiculitis, *n* (%)*	–	1 (2)	1	–
Additional clinical features:				
Optic nerve involvement, *n* (%)*	–	16 (26)	0.1	–
PNS involvement, *n* (%)	4 (40)	17 (28)	0.47	1.71 [0.31–8.3]
ICU admission, *n* (%)	4 (40)	18 (30)	0.49	1.58 [0.29–7.63]
Orotracheal intubation, *n* (%)*	–	10 (22)	0.33	–
mRS at presentation, median (range)	3 (2–5)	4 (1–5)	0.18	–
mRS > 2, *n* (%)	7 (70)	49 (80)	0.43	1.73 [0.25–9.13]
Abnormal initial brain MRI, *n* (%)	7 (70)	39 (64)	1	1.31 [0.26–8.65]
Abnormal initial spinal cord MRI, *n* (%)	4/8 (50)	20/34 (59)	0.71	0.71 [0.11–4.48]
CSF analysis:				
Protein (mg/dL), median (range)	97 (55–1830)	126 (24–500)	0.17	–
Elevated protein level (> 45 mg/dL), *n* (%)	9 (90)	57/59 (97)	0.38	0.32 [0.02–20.68]
White cell count (cell/μL), median (range)	270 (49–800)	270 (6–3740)	0.95	–
Elevated white cell count (> 5/μL), *n* (%) **	10 (100)	61 (100)	–	–
OCB (≥ 2), *n* (%)	6/9 (67)	27/33 (82)	0.38	0.45 [0.07–3.6]
Low glucose level (< 40 mg/dL), *n* (%) *	–	5 (9)	0.58	–
Follow-up CSF GFAP-IgG analysis, *n* (%)	4/7 (57)	13/22 (59)	1	0.93 [0.12–7.91]

*CI* confidence interval, *CSF* cerebrospinal fluid, *GFAP* glial fibrillary acidic protein, *ICU* intensive care unit, *IgG* immunoglobulin G, *MRI* magnetic resonance imaging, *mRS* modified Rankin scale, *OCB* oligoclonal bands, *OR* odds ratio, *PNS* peripheral nervous system

^a^Equivalent to the median age at onset

^*^Not possible to calculate OR since the event of interest (relapse) did not happen in one of the groups

^**^Not possible to calculate OR since the event of interest (relapse) happened in 100% of individuals in at least one of the groups

Bold value indicates the statistically significant parameters

**Table 5:** Survival analysis of the association of onset characteristics with relapse

	HR [95% CI]	*p* value
Male sex	1.64 [0.48–5.68]	0.4
Tumor	**4.55 [1.28–16.14]**	**0.03**
Clinical syndrome at first episode:		
Meningoencephalitis	1.13 [0.32–4.01]	0.8
Meningo-encephalo-myelitis	0.64 [0.14–3.02]	0.6
Meningitis	2.28 [0.48–10.87]	0.3
Myelitis*	–	–
Meningoradiculitis*	–	–
Additional clinical features:		
Optic nerve involvement*	–	–
PNS involvement	1.55 [0.44–5.49]	0.5
ICU admission	1.48 [0.42–5.27]	0.6
Orotracheal intubation*	–	–
Abnormal initial brain MRI	1.05 [0.27–4.05]	0.9
Abnormal initial spinal cord MRI	0.66 [0.16–2.63]	0.6
CSF analysis:		
Elevated protein level (> 45 mg/dL)	0.48 [0.06–3.77]	0.5
Elevated white cell count (> 5/μL)**	–	–
OCB (≥ 2)	0.45 [0.11–1.83]	0.3
Low glucose level (< 40 mg/dL)*	–	–
Follow-up CSF GFAP-IgG analysis	0.88 [0.2–3.98]	0.9

*CI* confidence interval, *CSF* cerebrospinal fluid, *GFAP* glial fibrillary acidic protein, *HR* hazard ratio, *ICU* intensive care unit, *IgG* immunoglobulin G, *MRI* magnetic resonance imaging, *OCB* oligoclonal bands, *PNS* peripheral nervous system

*Not possible to calculate HR since the event of interest (relapse) did not happen in one of the groups

**Not possible to calculate HR since the event of interest (relapse) happened in 100% of individuals in at least one of the groups

Bold value indicates the statistically significant parameters

## Discussion

In the present study, we found an overall good outcome at last follow-up, since more than three-quarters of patients had mRS ≤ 1 after a median follow-up of 28 months. Nevertheless, cognitive complaints, as well as gait and sphincter disorders were frequent, and around a third of those included could not return to work/school due to disability. Moreover, concomitant tumor at onset was associated with a higher rate of relapse.

Baseline clinical characteristics of the population reported herein are similar to that of published cases of GFAP, including sex ratio, age at onset and clinical presentation, with meningo-encephalitis with or without myelitis as the main phenotype at onset [4], and a severe presentation [12, 15–17]. Similarly, consistent with prior reports [9–12], the association with neoplasms was not frequent.

Recent studies focus on short-term outcome and find good recovery of the initial clinical episode and favorable course during follow-up [15, 16, 18], while long-term outcome of autoimmune GFAP astrocytopathy has not been exhaustively assessed. Herein, contrasting with initial severity (more than three-quarters had mRS ≥ 3 at diagnosis), after extended follow-up most patients had good functional outcome, since more than three-quarters had mRS ≤ 1 at last follow-up. Even though there was a clear dynamic towards minor sequela or even complete recovery, at last follow-up close to a quarter of patients had mRS ≥ 2. Nonetheless, more than half of the population reported symptoms or showed signs at the neurological examination leading to disability, such as gait disorders, cognitive complaints, sphincter disorders or visual impairment, and around one-third of the cohort could not return to work/school. Therefore, existing mild but still limiting neurological symptoms/signs during long-term follow-up are not to be neglected. These may lead clinicians to optimize management in order to achieve a complete recovery. Monitoring patients in a regular and prolonged manner after disease onset is essential to ensure recovery and return to professional and social activities.

Autoimmune GFAP astrocytopathy is mainly reported with a monophasic course [15, 16, 18]. In the present study, less than a fifth of patients relapsed, occurring mostly in the first year of the disease. In relapsing patients, disease control was achieved when the corticosteroid dose was increased or second-line immunoactive treatment was initiated, among which rituximab was the most frequently used. This observation may be consistent with the previously reported cases of cortico-dependence [1, 16] and may raise the consideration that corticosteroids should be prolonged. However, this could not be fully evaluated in the present study, and the association between early corticosteroid tapering/withdrawal and relapse should be further explored.

Concomitant tumor was associated with a higher risk of relapse. As mentioned above, autoimmune GFAP astrocytopathy is not considered as a paraneoplastic entity, even though diverse neoplasms have been reported [9–12]. Interestingly, this syndrome has been also described as a possible side effect of immune checkpoint inhibitors when used as a cancer treatment [19]. Nevertheless, the long-term prognostic impact of cancer on autoimmune GFAP astrocytopathy, unlike paraneoplastic neurological syndromes [20], has not been explored, although the presence of a tumor was associated with refractoriness to first-line immunotherapy [10]. In his context, the association of a tumor with future relapse found herein may have clinical implications, mainly cancer screening at initial presentation of autoimmune GFAP astrocytopathy and, if found, initiating early oncological treatment, but also considering long-term immunotherapy in such cases. No other risk factor for relapse was found in the present study, probably owing to the inclusion of few total relapsing-course patients. Due to the rarity of autoimmune GFAP astrocytopathy, larger multicenter studies should be conducted to further study relapse predictive factors.

Most brain and spinal cord MRI findings improved during follow-up, similarly to CSF analysis, thus demonstrating an overall course to regression of the initial inflammatory process. CSF GFAP-Abs analysis was monitored, and approximately, half of patients had a negative conversion after a median of 8 months. However, their persistence was not associated with a greater risk of relapse. Although it could not be evaluated herein, future research may focus on the role of persistent CSF GFAP-Abs on disease activity and relapsing phenotype, as this may have future implications for monitoring and treatment decisions. Despite some available emerging data [21], follow-up biomarkers of the disease are still lacking.

Regarding treatment strategy, acute treatment at onset is dispensed in most cases, in particular corticosteroid pulses but also IVIg infusions or plasma exchanges, generally resulting in a good short-term outcome [15, 16, 18]. However, the widespread use of acute therapies contrasts with less common administration of long-term treatment in patients with autoimmune GFAP astrocytopathy. Approximately, half of the patients included herein received at least one maintenance therapy at onset. Long-term oral corticosteroids were the most commonly administered, while other strategies were uncommon. Although treatment administration is heterogeneous in previous reports, oral corticosteroids are also the most commonly used long-term treatment and other long-term immunoactive treatments are administered in different combinations in a non-systematized manner [9, 10, 12, 13, 15, 16]. Thus, given the aforementioned long-term outcome, autoimmune GFAP astrocytopathy might have been undertreated when considering long-term in comparison to initial care. The awareness of the risk of relapse and the possibility of long-term disability raises the consideration that patients may probably benefit from long-term immunotherapy. We propose an optimization of the treatment algorithm, which may include oral corticosteroids with a slow and prolonged taper and additional second-line immunotherapy for at least 1 year, considering more aggressive or prolonged approach if an associated cancer is present. The therapeutic decision should be then reassessed in an individualized manner.

The main limitations of the present study are related to its retrospective nature and the loss of some patients during follow-up, causing limited sample size and potentially relevant missing data. In addition, due to the retrospective design, we were not able to systematically assess reported disabling symptoms at last visit, which may be particularly relevant for cognitive complaints. Lastly, GFAP-Abs testing was performed in patients registered in two French referral centers, causing a possible selection bias.

In conclusion, the present study suggests a greater impact than previously described in long-term functional outcome of patients with autoimmune GFAP astrocytopathy, despite a generally good outcome considering only the mRS. It confirms a mainly monophasic course but raises the possibility that relapses might be associated to early corticosteroids dose reduction, also outlining concomitant tumor at initial presentation as a risk factor for relapse.

## Supplementary Information

Below is the link to the electronic supplementary material.

Supplementary file1 (DOCX 13 KB)

## Data Availability

Any data not published within the article are available and will be shared by request from any qualified investigator.
