# Supplementary material for: Clinical course and long-term outcomes in autoimmune glial fibrillary acidic protein (GFAP) astrocytopathy
Source: J Neurol. 2025 May 26;272(6):421. doi: 10.1007/s00415-025-13159-0 (PMC12106124; doi:10.1007/s00415-025-13159-0)
Supplement: Supplementary file 1 — Supplementary file1 (DOCX 13 KB) [file 415_2025_13159_MOESM1_ESM.docx]

**Table 1.** Demographic and clinical characteristics of deceased patients during follow-up.

| **Patients** | **Sex**  **(M/F)** | **Tumor** | **Clinical syndrome at onset** | **mRS**  **at presentation** | **ICU admission** | **Long-term treatments** | **Follow-up (months)** | **Cause of death** |
| --- | --- | --- | --- | --- | --- | --- | --- | --- |
| **No. 1** | M | Renal carcinoma | 1.Meningoencephalitis | 5 | No | OCS taper | 15 | Status epilepticus and respiratory complications secondary to brain metastasis |
|  |  |  | 2. Meningoencephalitis  (4 months later) | 5 | No | OCS  Chemotherapy |  |  |
| **No. 2** | F | No | Meningo-encephalo-myelitis | 5 | Yes, with intubation | OCS  Rituximab | 15 | Progressive leukopathy and aspiration pneumonia |
| **No. 3** | M | No | Meningoencephalitis | 6 | Yes, with intubation | No | 2 | Refractory status epilepticus and intracranial hypertension |

F: female, ICU: intensive care unit, M: male, mRS: modified Rankin scale, OCS: oral corticosteroids.
